# Editorial: Artificial intelligence for personalized and predictive genomics data analysis

**DOI:** 10.3389/fgene.2023.1162869

**Published:** 2023-03-03

**Authors:** Zeeshan Ahmed, Saman Zeeshan, Donghyung Lee

**Affiliations:** ^1^ Rutgers Institute for Health, Healthcare Policy and Aging Research, Rutgers University, New Brunswick, NJ, United States; ^2^ Department of Medicine, Robert Wood Johnson Medical School, Rutgers Biomedical and Health Sciences, New Brunswick, NJ, United States; ^3^ Rutgers Cancer Institute of New Jersey, New Brunswick, NJ, United States; ^4^ Department of Statistics, Miami University, Oxford, OH, United States

**Keywords:** artificial intelligence, genomics, personalized medicine, predictive analysis, machine learning

The quest to understand what causes chronic, acute, infectious, and rare diseases has been a central focus of human health studies since the beginning of scientific discovery (Ahmed, 2020). Our evolving understanding of their complex nature has led us to realize the importance of effective diagnosis and treatment of patients with these conditions (Ahmed et al., 2020). Over the last few decades, genomics has been leading us towards an audacious future; it has been changing our views about conducting biomedical research, studying diseases, and understanding diversity in our society across the human species (Zeeshan et al., 2020). However, there are more unknowns than knowns in genomics. By identifying the novel risk factors and disease biomarkers, genomics and precision medicine has the potential to translate scientific discovery into clinically actionable personal healthcare (Ahmed, 2022). Nevertheless, we still require innovative and intelligent solutions to advance genomics and precision medicine, such as creating new models of medicine where physicians use clinical decision support systems based on Artificial Intelligence (AI) and Machine Learning (ML) to choose the best treatment for a patient guided by the genomics variants that each of us has (Vadapalli et al., 2022).

The rightful use of the AI/ML can accelerate our ability to leverage and impact the overall way of scientific research. This Research Topic focuses on the publication of AI/ML approaches proposed to facilitate the implementation of genomics and precision medicine and accelerate diagnostic and preventive care delivery strategies that go beyond traditional symptom-driven, disease-causal medical practice. Successfully achieving the goals of this Research Topic, we were able to publish five interesting peer-reviewed articles, addressing variable disease-specific challenges among diverse populations and providing valuable insights to direct future research based on AI/ML techniques.

In, “*SPCMLMI: A structural perturbation-based matrix completion method to predict lncRNA–miRNA interactions*”, Wang et al. have discussed the significance, methodology, and implementation of their computational model, i.e., SPCMLMI, for inferring lncRNA-miRNA interactions through a structural perturbation-based matrix completion. In this study, authors evaluated the prediction performance of this novel model and exercised it to infer potential interactions of lncRNAs with miRNAs. In addition, to justify the strengths of SPCMLMI, they compared it with the other three related networks.

In, “*Development and validation of a chromatin regulator prognostic signature in colon adenocarcinoma*”, Yang et al. presented their recent research to investigate chromatin regulators, including differentially expressed genes, regulation network, correlations, and gene alterations in colon adenocarcinoma (COAD). To achieve the goals of their study, they conducted a clustering analysis to identify the molecular subtypes in COAD. The overall methodology discussed in this important study included the implementation and validation of a prognostic model based on chromatin regulators in COAD; the development of a nomograph scoring tool for predicting individual prognosis outcomes; and the analysis of enriched pathways. Yang et al. concluded with two molecular subtypes in COAD using chromatin regulators, and the potential to provide new risk management and individualized treatment strategies for COAD.

Zheng introduced an interesting study, “*TLsub: A transfer learning based enhancement to accurately detect mutations with wide-spectrum sub-clonal proportion*”, where the author proposed a method to deconstruct the relationship between sequencing signals and the clonal proportion by analyzing signal data. In this study, Zheng employed a transfer learning method to reconstruct a new reproducing Hilbert space and filtering false positive calls to accurately detect mutations with wide spectrum subclonal proportion.

In, “*Molecular subtypes based on cuproptosis regulators and immune infiltration in kidney renal clear cell carcinoma*”, Liu et al. presented their perspective on the functional roles of cuproptosis in kidney renal clear cell carcinoma (KIRC), and provided insight for the development of an individualized treatment strategy. Authors were focused on investigating gene alterations and variations in the number of cuproptosis regulators genes in KIRC. They explored the clinical and immune characteristics with a clustering analysis based on differentially expressed cuproptosis regulators. Liu et al. implemented and validated their prognostic model by calculating a risk score to predict the prognosis of the individual patient using a novel nomogram risk assessment model.

In, “*Artificial Intelligence, Healthcare, Clinical Genomics, and Pharmacogenomics Approaches in Precision Medicine*”, Abdelhalim et al. highlighted the significance of various fields, including healthcare, genomics, clinical genomics, and pharmacogenomics in precision medicine. They discussed the improvements, which could be made to these fields with the use of AI, ML, and big data, especially for the development and provision of precision medicine.

The publication of our Research Topic, with the inclusion of all these important articles, will potentially contribute to the sharing of impactful literature based on quality research and development, involving AI/ML techniques for the personalized and predictive genomics data analysis.
